# Repetitive Transcranial Magnetic Stimulation with and without Internet-Delivered Cognitive Behavior Therapy for the Treatment of Resistant Depression: Patient-centered Randomized Controlled Pilot Trial

**DOI:** 10.1192/j.eurpsy.2023.897

**Published:** 2023-07-19

**Authors:** M. K. Adu, R. Shalaby, E. Eboreime, A. Sapara, P. Chue, A. Greenshaw, V. Agyapong

**Affiliations:** 1 Psychiatry, Dalhousie University, Halifax NS, Halifax; 2 Psychiatry, University of Alberta, Edmonton, Canada

## Abstract

**Introduction: Background:**

Treatment-resistant depression (TRD) is considered one of the major clinical challenges in the field of psychiatry. At least 15% of all patients with MDD remain refractory to any treatment intervention. Repetitive transcranial magnetic stimulation (rTMS) is considered a treatment option for patients with TRD. Additionally, iCBT is an evidence-based psychotherapy for the management of TRD.

**Objectives:**

This study aims to evaluate the clinical effectiveness of adding iCBT to rTMS treatment as an innovative combined intervention, exploring the short and long-term outcomes on patients with TRD

**Methods:**

This study is a randomized controlled trial. Participants diagnosed with TRD were randomized to one of two interventions: rTMS alone and rTMS+iCBT. Each group completed evaluation measures at baseline, discharge (6 weeks), and one & three months after discharge. The primary outcome measure was the mean change in the Hamilton depression rating scale (HAMD-17) from baseline to three months.

**Results:**

Preliminary results for the early outcome of the study showed that after adjusting for the baseline scores, there was no significant difference in the mean score of HAMD-17 from baseline to six weeks between the participants of the two groups, (F (1, 53) = 0.15, p = 0.70, partial eta square = 0.003). The result of the long-term effectiveness is underway, forecasting the potential synergism of the two interventions.

**Conclusions:**

This study found the combined treatment of rTMS + iCBT not to be superior to treatment with rTMS alone, in the short term. We hope the long-term results would thoroughly address the effectiveness of the combined therapy in this randomized controlled trial.

**Disclosure of Interest:**

None Declared

